# The diagnostic and therapeutic challenges of pulmonary epithelioid trophoblastic tumor: A case report and literature review

**DOI:** 10.1097/MD.0000000000048248

**Published:** 2026-04-10

**Authors:** Zhuo Yang, Shufang Chang, Jiangchuan Sun, Qianqian Wang

**Affiliations:** a Department of Obstetrics and Gynecology, The Second Affiliated Hospital of Chongqing Medical University, Chongqing, China.

**Keywords:** epithelioid trophoblastic tumor, fertility preservation, gestational trophoblastic neoplasm, lung cancer, misdiagnosis

## Abstract

**Rationale:**

Primary pulmonary epithelioid trophoblastic tumor (ETT) without a uterine primary is exceptionally rare, with merely 29 cases documented in the English-language literature. Therefore, a standardized management protocol has yet to be established, and the optimal treatment strategy remains poorly defined.

**Patient concerns:**

A 31-year-old woman presented to a local hospital with irregular vaginal bleeding and was initially diagnosed with an ectopic pregnancy due to elevated serum beta-human chorionic gonadotropin (β-hCG) levels. Following treatment with diagnostic curettage and methotrexate, her β-hCG levels plateaued. The patient was subsequently referred to our institution for further investigation. Imaging revealed a 1.7 cm × 1.5 cm pulmonary nodule, which was initially suspected to be peripheral lung cancer.

**Diagnoses:**

Video-assisted thoracoscopic resection and subsequent histopathological analysis confirmed the diagnosis of ETT.

**Interventions:**

The patient received 4 cycles of combined EMA/EP (etoposide, methotrexate, actinomycin D/etoposide, cisplatin) chemotherapy after the surgery.

**Outcomes:**

During 3 years of follow-up, her menstrual cycles were normal and there was no evidence of disease recurrence.

**Lessons:**

This case highlights the diagnostic challenges and management strategies for pulmonary ETT, particularly in preserving fertility while ensuring optimal oncological outcomes.

## 1. Introduction

The epithelioid trophoblastic tumor (ETT) is a rare type of gestational trophoblastic neoplasm originating from intermediate trophoblastic cells of the chorion laeve. It primarily affects women of reproductive age, with the uterine corpus being the most common site of origin, followed by the cervix. Although ETT can metastasize to various sites, including the lungs,^[1]^ small bowel, fallopian tube, broad ligament, and skin,^[2]^ pulmonary ETT in the absence of a uterine lesion is exceptionally rare, with only 29 cases documented in the English-language literature to date. This condition is frequently subject to misdiagnosis and delayed detection, and the absence of established treatment guidelines contributes to ongoing uncertainty regarding its optimal management.

We present the case of a 31-year-old woman who was initially misdiagnosed with an ectopic pregnancy due to amenorrhea, vaginal bleeding, and elevated serum β-hCG levels. The diagnosis of pulmonary ETT was ultimately confirmed following surgical resection of a pulmonary nodule. By reporting this case and providing a comprehensive review of the literature – with particular focus on treatment strategies and considerations for future pregnancy – we aim to contribute to the limited evidence base surrounding this rare clinical entity.

## 2. Case report

A 31-year-old woman (gravida 3, para 1) presented to a local hospital with a 17-day history of irregular, scant vaginal bleeding. Her obstetric history was unremarkable, with the most recent pregnancy terminated by induced abortion 12 months prior. Pelvic examination was normal. The initial serum β-hCG level was 304 mIU/mL. Given 49 days of amenorrhea and transvaginal ultrasonography showing an 8 mm endometrial thickness without an intrauterine or adnexal gestational sac, a provisional diagnosis of ectopic pregnancy was established. Diagnostic curettage yielded proliferative-phase endometrium. Postoperatively, the serum β-hCG levels rose to 500 mIU/mL, prompting administration of intramuscular methotrexate (50 mg/m^2^ on days 1 and 4) for suspected ectopic pregnancy. By day 11, the β-hCG level declined to 370 mIU/mL (a reduction of >15%), and the patient was discharged for outpatient follow-up. However, over the subsequent 2 months, weekly serum β-hCG monitoring showed a plateau, with fluctuations between 349 and 561 mIU/mL. A diagnosis of persistent ectopic pregnancy was considered, although repeat transvaginal ultrasonography failed to identify a gestational focus. Given the patient’s stable hemodynamic status, surgical intervention was deferred, and she was managed conservatively with oral mifepristone 50 mg and adjunctive traditional Chinese medicine. She remained asymptomatic, reporting no abdominal pain, respiratory symptoms, or chest discomfort. Nonetheless, her menstrual cycles did not resume, and she experienced an additional episode of minor vaginal bleeding 20 days after discharge.

The patient was referred to our institution for further evaluation of persistently low but detectable serum β-hCG levels. On admission, her serum β-hCG level was 349.20 mIU/mL. Transvaginal ultrasonography revealed an endometrial thickness of 12 mm. A positive urine pregnancy test confirmed circulating β-hCG, excluding assay-related false positivity. The differential diagnosis included persistent ectopic pregnancy and quiescent gestational trophoblastic disease.

**Figure 1.**
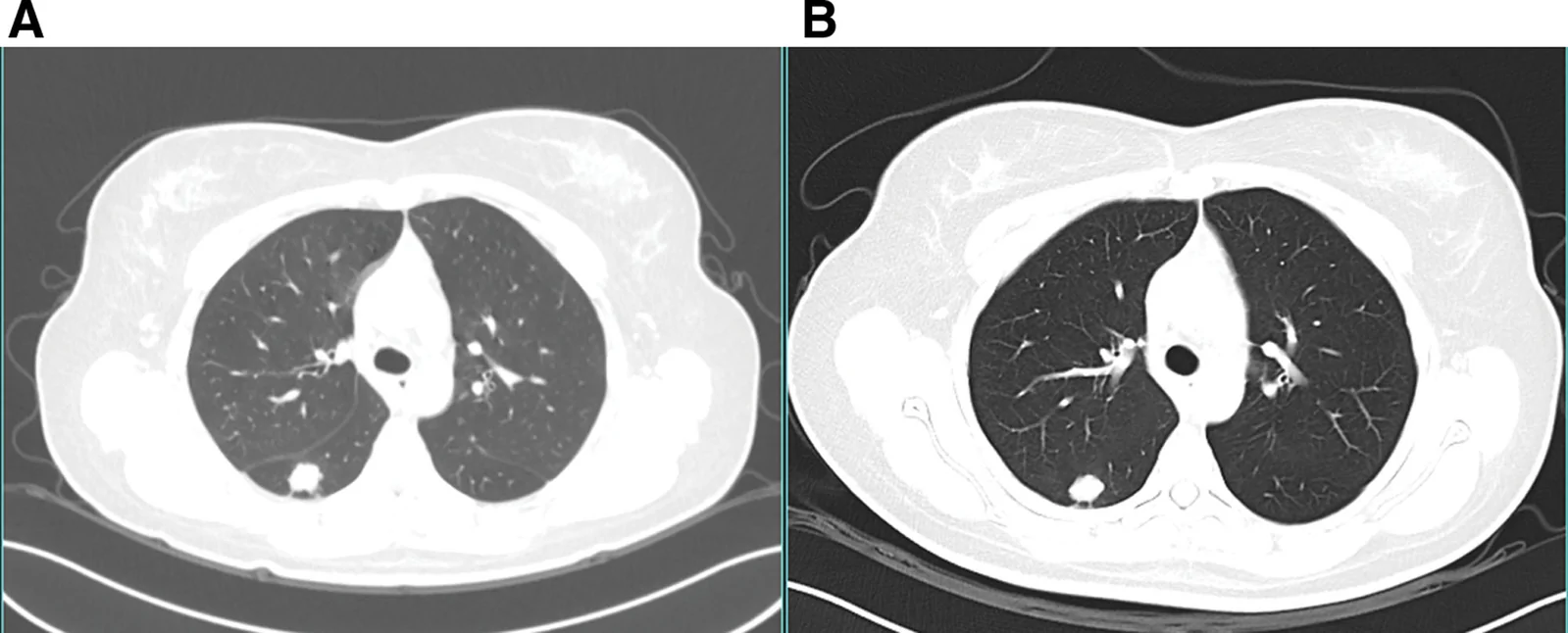
(A) A CT scan of the chest revealed a 1.7 cm × 1.5 cm solid nodule in the dorsal segment of the right lower lobe, with burrs and lobes on edge, and the adjacent pleura was stretched and thickened. (B) Contrast-enhanced CT scan showed heterogeneous enhancement. CT = computed tomography.

A treatment plan was formulated, involving a second course of methotrexate therapy (75 mg, intramuscular) with close monitoring of serum β-hCG levels. Additionally, computed tomography (CT) of the chest and abdomen was conducted to identify potential sources of ectopic β-hCG secretion. CT imaging revealed a 1.7 cm × 1.5 cm nodule in the dorsal segment of the right lower lung lobe (Fig. 1), raising suspicion of peripheral lung cancer. Given the possibility of a paraneoplastic syndrome, a thoracic surgery consultation was obtained. The patient denied respiratory symptoms such as cough, chest pain, or hemoptysis. Lung cancer biomarkers were within normal limits, and a tuberculin test was negative. Positron emission tomography–computed tomography (PET-CT) demonstrated increased 18-fluorodeoxyglucose (FDG) uptake in the lung nodule, suggestive of malignancy. The patient subsequently underwent thoracoscopic nodule resection. Intraoperative frozen section analysis indicated malignancy, leading to right upper lobectomy and lymph node dissection.

**Figure 2.**
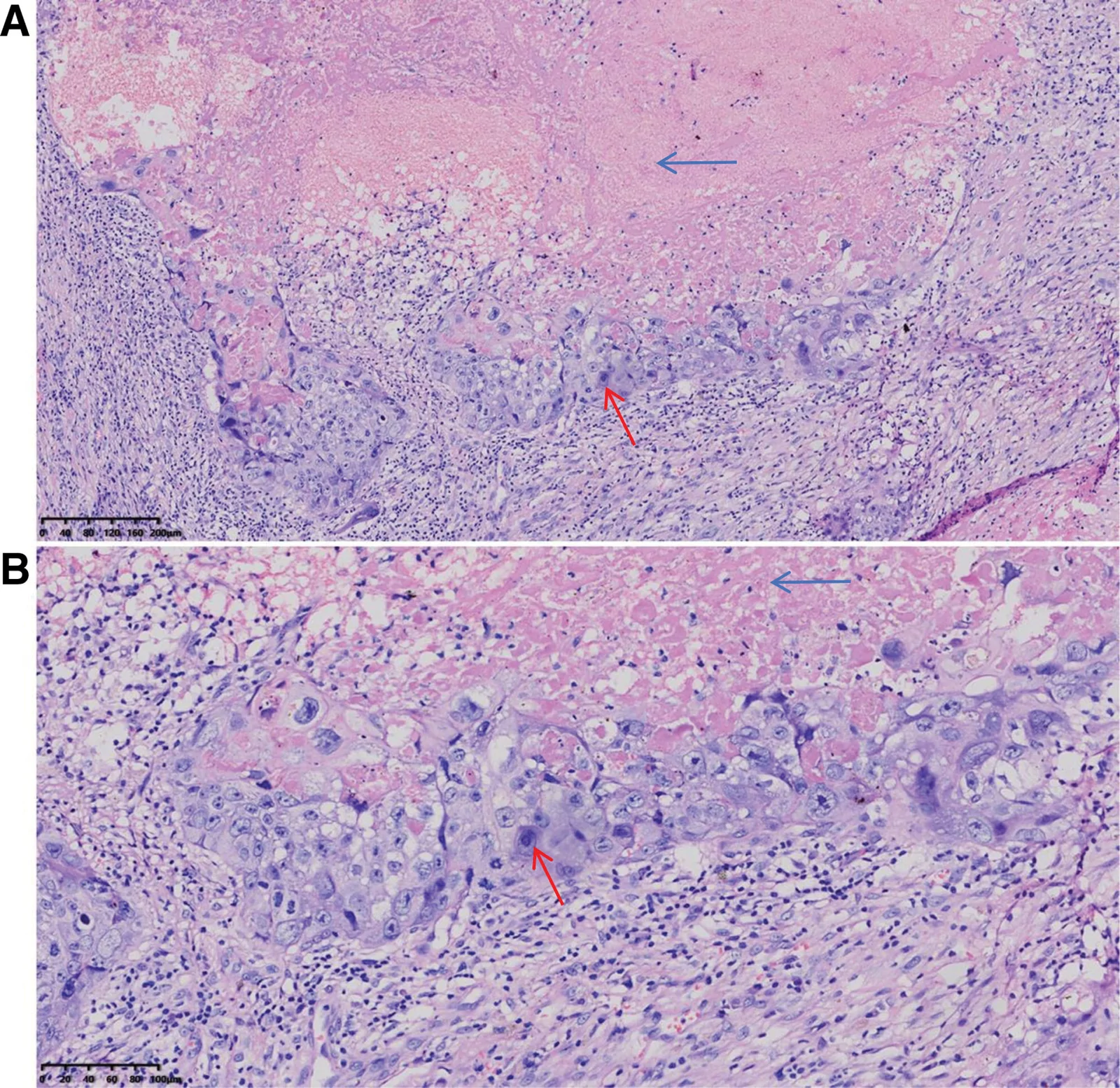
Microscopic examination. The tumor tissue appears as single trophoblast monocyte arranged in nests or cords, with necrosis of a cellulose-like transparent substance (hematoxylin-eosin staining, red arrow: trophoblast nests; blue arrow: necrosis, A, ×10; B, ×20).

**Figure 3.**
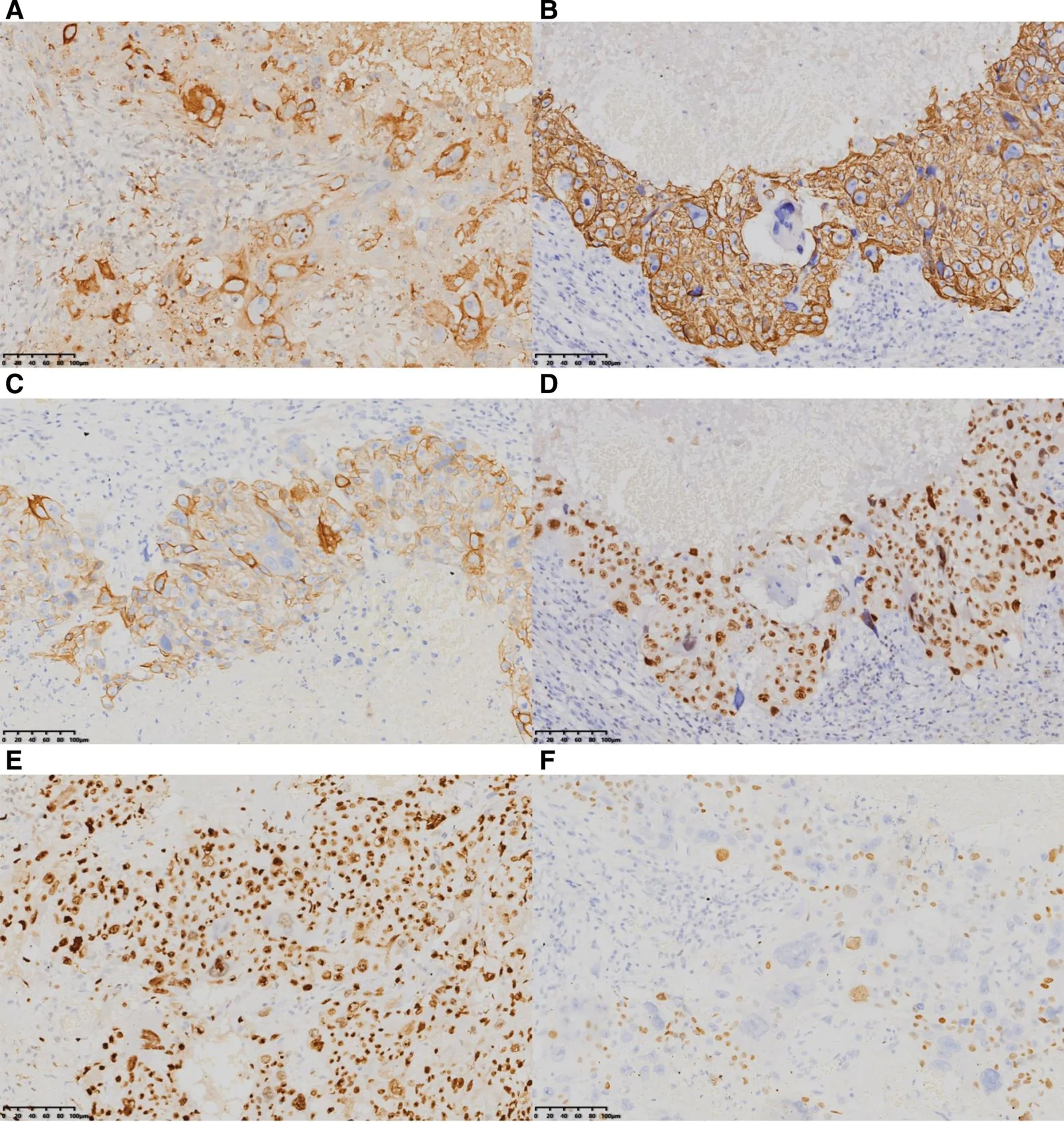
Immunohistochemical staining (×20). (A) HCG (+); (B) CK18 (++); (C) CK7 (+); (D) GATA-3 (++); (E) Ki-67 (+) 80%; (F) P63 (+).

**Figure 4.**
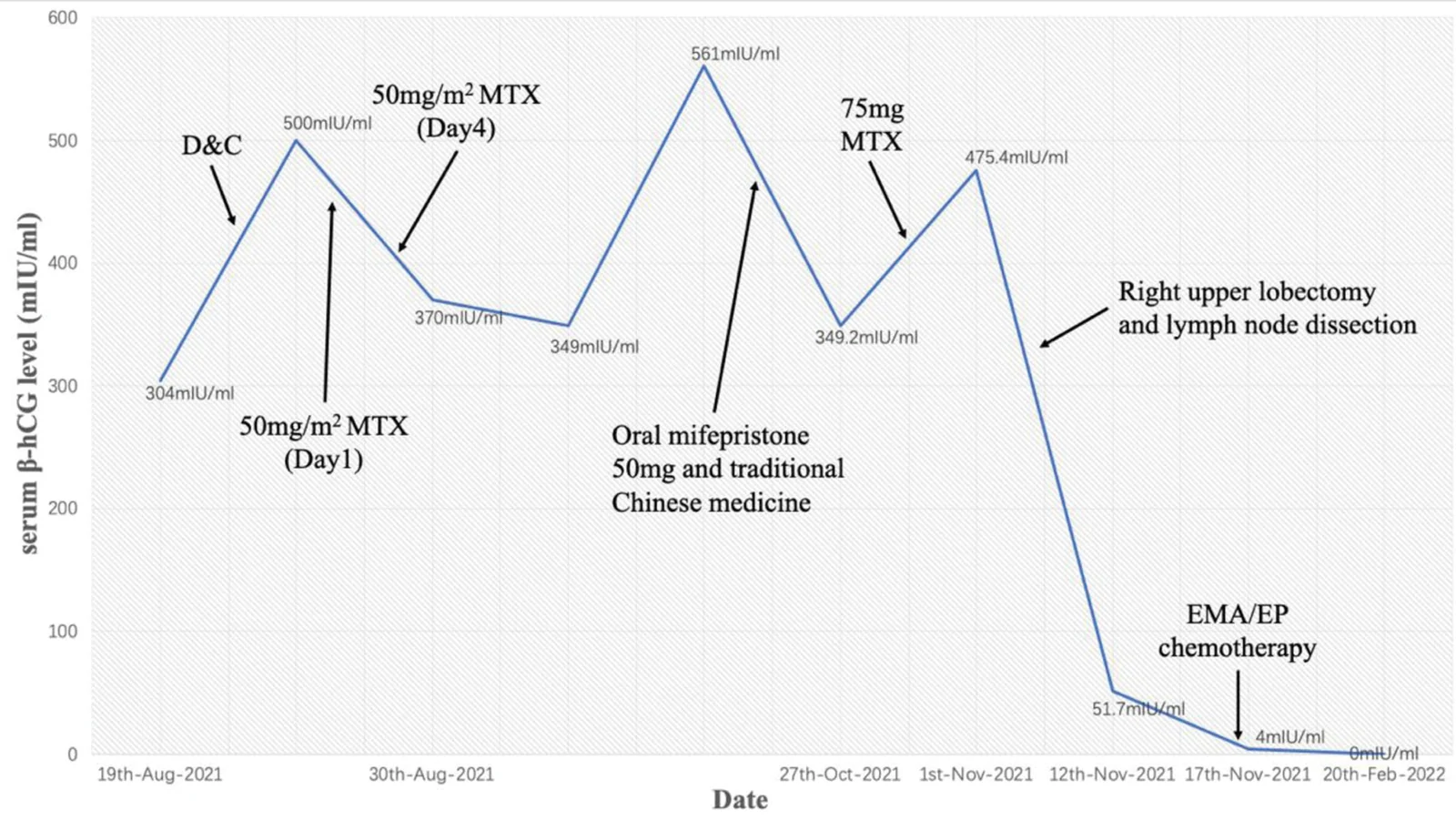
Curve showing serum β-hCG level during the diagnosis of ETT and in response to the treatment procedures. β-hCG = beta-human chorionic gonadotropin, EMA = etoposide, methotrexate and actinomycin D, EP = etoposide, cisplatin, ETT = epithelioid trophoblastic tumor, MTX = methotrexate.

Histopathological and immunohistochemical analyses of the lung tissue confirmed ETT, showing positivity for CK, Ki-67 (80%), HCG, P63, CK7, CK18, and GATA-3, and negativity for α-inhibin, PLAP, CK20, GPC-3, HPL, and EMA (Figs. 2 and 3). Three regional lymph node groups showed no metastatic involvement. On the third day after the operation, the β-hCG level dropped to 51.76 mIU/mL (Fig. 4). Whole-body PET-CT revealed no evidence of intrauterine or distant metastatic disease. In light of absent uterine involvement and the patient’s desire for fertility preservation, hysterectomy was not performed. She received 4 cycles of combination EMA/EP chemotherapy (etoposide, methotrexate, actinomycin D, followed by etoposide and cisplatin), and the serum β-hCG level dropped to normal (<0.001 mIU/mL). At the 3-year posttreatment follow-up, the patient reported regular menstrual cycles, with serum β-hCG levels consistently within normal limits. Surveillance imaging – biannual chest CT (Fig. S1, Supplemental Digital Content, https://links.lww.com/MD/R622) and pelvic ultrasound – showed no evidence of recurrence.

## 3. Discussion

Pulmonary ETT is a rare subtype of gestational trophoblastic tumor (GTN), and its pathogenesis remains incompletely understood. DNA sequencing identified paternal Y-chromosome loci and novel alleles in pulmonary lesions, providing molecular evidence for a fetal (placental) rather than maternal origin.^[3]^ Research indicates that trophoblastic cells may persist in the maternal pulmonary circulation during the peripartum period, raising the possibility that GTN presenting initially in the lungs may arise from de novo transformation of trophoblast embolized to the pulmonary tissue during a prior pregnancy.^[4]^ Alternatively, the primary uterine lesion may be too small or indolent to be detected by current imaging techniques, or it may have undergone spontaneous regression following metastatic spread.^[5]^ A review of isolated pulmonary GTN indicates that ETT is the most common histologic subtype, accounting for 29 out of 40 reported cases.^[6]^ However, the biological basis for this predominance remains unclear. Owing to its extreme rarity, pulmonary ETT is often underrecognized in clinical practice, posing considerable diagnostic and management challenges. In an effort to address this knowledge gap, we present the current case alongside a comprehensive review of the literature, with the aim of improving the understanding and clinical approach to this unusual form of GTN.

A systematic review of English-language literature identified 29 reported cases of pulmonary ETT with clinical features comparable to those of the present case, all documented in isolated case reports or small case series (Table 1).^[7-19]^ The mean age at diagnosis was 33.7 years (range: 23–49). The most common presenting symptom was vaginal bleeding or abnormal menstruation, reported in 34.5% (10/29) of patients. In contrast, respiratory symptoms such as cough, hemoptysis, or pleuritic chest pain were observed in only 10.3% (3/29) of cases. A subset of patients (7/29) were asymptomatic, with lesions identified incidentally during routine radiographic imaging or laboratory examination. Obstetric history varied widely, with antecedent pregnancies including term deliveries (n = 10), abortions (n = 4), hydatidiform moles (n = 4), and invasive moles (n = 1). The interval between the most recent pregnancy and ETT diagnosis ranged from 3 to 96 months, underscoring the variable latency period of the disease. Serum β-hCG elevation was observed in 86.2% (25/29) of cases, with levels remaining below 2500 mIU/mL (median: 493 mIU/mL). Among the 25 cases with available imaging data, the mean maximum diameter of pulmonary nodules was 3.12 cm (range: 0.8–7.5 cm). All patients underwent surgical resection of the pulmonary lesion, and no lymph node metastasis was reported. Postoperative management varied, with 6 patients (20.7%) undergoing hysterectomy and thirteen (44.8%) receiving adjuvant chemotherapy. Disease recurrence occurred in 3 patients (10.3%), manifesting as isolated pulmonary nodules within 6 months of initial treatment; all 3 achieved complete remission following secondary surgical resection. During a follow-up period ranging from 3 to 90 months, no distant metastases or disease-related mortality were reported.

**Table 1:** Clinical features of pulmonary ETT cases.

Authors	Patient no.	Age (yr)	Symptoms	Preceding pregnancy	Interval from last pregnancy, mo	Provisioal diagnosis	Number of metastases in lungs	Pretreatment hCG, mIU/mL	Maximum diameter of tumor (cm)	Surgical resection of pulmonary lesion	Hysterectomy	Preoperative chemotherapy	Postoperative chemotherapy	Initial remission, mo	Recurrence	Subsequent treatment	Second remission, mo	Follow-up, survival, mo
Lei et al^[7]^	1	49	None	Hydatidiform mole	NA	Lung cancer	1	NA	3	Thorascopic lower left lobectomy with mediastinal lymphadenectomy	−	−	−	3	−	/	/	3
Urabe et al^[8]^	2	38	Poor physical condition	Term pregnancy	12	NA	Multiple	80.1	0.8	Thorascopic right segmentectomy	−	6 cycles of EMA-CO	+	6	+	Surgical resection of pulmonary lesions	3	9
Shih et al.^[5]^	3	42	Hemoptysis and cough	Term pregnancy	NA	Lung cancer	1	1300	5	VATS	−	−	Several courses after chemotherapy		−	/	/	24
Li et al^[9]^	4	31	None	Subclinical miscarriage	48	NA	1	168.1	1	Thorascopic left upper lobe segmentectomy	−	−	3 cycles of EP-EMA	13	−	/	/	13
Okereke et al^[10]^	5	40	Vaginal bleeding	NA	NA	Non-small cell lung cancer	1	1100	6	Thorascopic lobe lobectomy with mediastinal lymph node dissection	−	−	4 cycles of cisplatin/etoposide	12	−	/	/	12
Kim et al^[11]^	6	35	Abdominal pain, nausea, and vomiting	Term pregnancy	NA	SCLC	1	NA	3.3	Thorascopic left lower lobectomy with mediastinal lymph node dissection	−	−	−	15	−	/	/	15
Ahn et al^[12]^	7	26	Delayed and relatively heavy menstruation	Suspected subclinical miscarriage	48	Lung cancer	1	NA	7.5	Thorascopic right lower lobectomy with mediastinal lymph node dissection	−	−	6 cycles of EMA/CO	9	−	/	/	9
Abro et al^[13]^	8	31	Irregular vaginal bleeding	Term pregnancy	96	Miscarriage, NSCLC	1	700	5	Thorascopic right lower lobectomy and systematic mediastinal lymphadenectomy	−	−	−	12	−	/	/	12
Sobecki-Rausch et al^[14]^	9	28	None	Term pregnancy	48	Ectopic pregnancy	1	50	1.7	Thorascopic wedge resection of the right lower lobe	+	−	3 cycles of TP/TE	47	−	/	/	47
Fenichel et al^[3]^	10	29	Vaginal bleeding	Term pregnancy	48	Ovarian β hCG-secreting germ-cell tumor, NSCLC	1	250	0.8	Unilateral ovariectomy, thorascopic left superior lobectomy	−	−	6 cycles of EP-EMA	12	−	/	/	12 (vaginal delivery after a year)
Huang et al^[15]^	11	32	Cessation of menstruation, vaginal bleeding, chest pain	Term pregnancy	24	Ectopic pregnancy	3	75	2.4	Right upper wedge resection,Right lower segmentectomy and lymph node dissection	−	75 mg MTX	−	1	+	Left pulmonary lesion resection	12	12
Lewin et al^[16]^	12	38	None	Term pregnancy	42	Large cell LC	1	400	2	Thorascopic right wedge resection and lobectomy	−	−	−	90	−	/	/	90
	13	49	Vaginal bleeding	Miscarriage	12	LCA	1	2204	6.2	Right lower lobectomy with mediastinal lymph node dissection	+	3.5 cycles of EMA/EP	−	45	−	/	/	45
	14	34	Irregular menses	Term pregnancy	24	NA	1	426	3	Right upper lobe segmentectomy	+	4 cycles of MTX	3 cycles of EMA/EP	22	−	/	/	22
Li et al^[17]^	15	28	Vaginal bleeding	Molar pregnancy	17	GTN	1	2021	NA	VATS in right lung	−	5 cycles of TP	2 cycles of EP-EMA + 5 cycles of EP	NA (but remission)	−	/	/	NA
	16	23	None	Abortion	11	Ectopic pregnancy	1	227	NA	VATS	+	−	3 cycles of EP-EMA	NA (but remission)	−	/	/	NA
	17	32	Vaginal bleeding, cough	Term pregnancy	3	Lung cancer	1	194	3.1	VATS	+	−	3 cycles of EP-EMA	NA (but remission)	−	/	/	NA
	18	30	Vaginal bleeding	Molar pregnancy	14	GTN	1	144	NA	VATS	−	−	3 cycles of EMA-CO		−	/	/	NA
Hamazak et al^[18]^	19	47	None	Invasive mole	36	Lung metastasis of breast cancer	1	0.7	NA	Thorascopic right upper lung lesion resection	For invasive mole 3 years ago	−	−	24	−	/	/	24
	20	32	None	Molar pregnancy	60	Lung cancer	1	NA	3	Right upper lobectomy	−	−	−	36	−	/	/	36
	21	42	Hemoptysis and cough	NA	NA	Lung cancer	1	1300 (after operation)	5	Left upper lobectomy and lymph node dissection	−	−	+	24	−	/	/	24
Liu et al^[19]^	22–27*	Average 30.5 (23–37)	NA	NA	Averagr 18 (3–77)	NA	NA	Average 213 (30.5–2247.7)	Average 2.4 (0.9–3.2)	Pulmonary lesion resection	−	NA	NA	NA (but remission)	−	/	/	30.5 (median)
	28		NA	NA	NA	CCA or IM				Pulmonary lesion resection	+	+	−	6	+	Pulmonary lesion resection and hysterectomy	NA	/
	29									Pulmonary lesion resection	−	+	−	NA (but remission)	−	/	/	/

+ = positive, − = negative, / = blank. CCA = choriocarcinoma, CO = cyclophosphamide, vincristine, EMA = etoposide, methotrexate and actinomycin D, EP = etoposide, cisplatin, ETT = epithelioid trophoblastic tumor, GTN = gestational trophoblastic neoplasia, hCG = human chorionic gonadotropin, LC = lung cancer/lung carcinoma, MTX = methotrexate, NA = not available, NSCLC = non-small cell lung cancer, PTNB = percutaneous transthoracic needle biopsy, TE = docetaxel, epirubicin, TP = paclitaxel, cisplatin, TS = thoracic surgery, VATS = video-assisted thoracoscopic surgery.

* Data for cases 22 to 27 are derived from Liu W et al (Front Oncol 2022). They are presented in their original aggregated format in this table due to data availability constraints.

Current evidence indicates that pulmonary ETT lacks specific clinical or radiological manifestations, posing considerable diagnostic challenges. Similar to the present case, 3 previously reported patients were initially misdiagnosed with ectopic pregnancy due to abnormal uterine bleeding, menstrual irregularities, and mildly elevated serum β-hCG levels. In all documented cases, pulmonary lesions were first identified by CT. Radiologically, these nodules are indistinguishable from primary lung malignancies. Moreover, the possibility of ectopic β-hCG production by certain lung cancers further complicates the clinical picture, often leading to an initial misdiagnosis of primary pulmonary malignancy. Definitive diagnosis consistently relies on postoperative histopathological and immunohistochemical confirmation of ETT.

The optimal management of pulmonary ETT without uterine involvement remains a subject of ongoing debate. According to the 2025 National Comprehensive Cancer Network (NCCN) Clinical Practice Guidelines, surgical intervention remains the cornerstone of treatment for ETT. For patients with disease confined to the uterus, the standard of care involves total hysterectomy with bilateral salpingectomy, with or without pelvic lymph node sampling. In cases of metastatic spread, this surgical approach is supplemented with multi-agent chemotherapy. For isolated distant metastases, particularly in the lungs, complete surgical resection is recommended when anatomically and clinically feasible.^[20]^ Notably, available case series and retrospective analyses have not demonstrated a clear survival benefit for hysterectomy in pulmonary ETT. Histopathological examination of resected uterine specimens has consistently shown no evidence of residual or concurrent intrauterine disease.^[15]^ Furthermore, in the 3 reported cases of pulmonary ETT recurrence, all relapses occurred as isolated pulmonary nodules without gynecological or extrathoracic spread, and all patients achieved complete remission after repeat surgical resection. These observations support the feasibility of fertility-sparing treatment strategies in patients with completely resectable pulmonary ETT, particularly in younger women who wish to preserve reproductive potential. Preliminary outcomes suggest that such an approach may achieve oncologic outcomes comparable to more radical interventions, as reflected by comparable disease-free survival rates (100% vs 100%), while reducing the impact on fertility and quality of life.

Epithelioid trophoblastic tumor exhibits relative resistance to chemotherapy, and the role of adjuvant systemic treatment after complete pulmonary resection remains controversial. According to NCCN guidelines, chemotherapy is recommended for patients with stage I disease exhibiting high-risk features, as well as for all individuals with metastatic involvement. Poor prognostic indicators include: an interval of ≥4 years since the antecedent pregnancy, deep myometrial invasion, extensive tumor necrosis, and elevated mitotic activity (>5 mitoses per 10 high-power fields). Preferred multi-agent chemotherapy regimens for metastatic ETT include EMA/EP (etoposide, methotrexate, actinomycin D/etoposide, cisplatin), EP/EMA, and TP/TE (paclitaxel/cisplatin and paclitaxel/etoposide) protocols, typically administered every 2 weeks, with 3 to 4 consolidation cycles after hCG normalization.

Limited retrospective evidence suggests that pulmonary ETT generally follows an indolent course, with outcomes comparable to stage I uterine ETT.^[19]^ This observation has led to divergent views on its management. Some experts advocate classifying pulmonary ETT as stage I disease, arguing that adjuvant chemotherapy may be omitted in patients – particularly younger ones – who achieve prompt hCG normalization after complete resection, thereby avoiding treatment-related toxicities such as gonadotoxicity, secondary malignancies, and long-term organ dysfunction. In contrast, others support classifying it as stage III disease due to pulmonary involvement, thus justifying adjuvant chemotherapy.^[14]^ Among the patients who did not undergo hysterectomy but received chemotherapy, 92.3% (8/9) achieved sustained complete remission. One patient with multifocal pulmonary involvement experienced disease recurrence following initial pulmonary resection and chemotherapy but subsequently achieved remission after secondary surgical intervention. No severe chemotherapy-related complications have been consistently documented across the cohort. Six patients underwent pulmonary lesion resection without chemotherapy and remained recurrence-free during the follow-up period. Given the limited cohort size, the long-term safety of a chemotherapy-free approach cannot be established. Nevertheless, the current body of evidence has not shown adjuvant chemotherapy to confer a statistically significant improvement in overall survival or recurrence risk for pulmonary ETT.

In the present case, adjuvant chemotherapy was administered based on 2 primary considerations. First, the tumor exhibited high proliferative activity, as evidenced by a Ki-67 index of 80%, which is associated with an increased risk of recurrence. Second, prevailing pathogenic models – namely, de novo malignant transformation of embolized trophoblastic cells from a prior gestation or hematogenous dissemination from an occult or regressed uterine lesion – suggest the possibility of residual circulating tumor cells or premalignant trophoblasts persisting after surgical resection. Therefore, adjuvant chemotherapy was employed to target potential micrometastatic disease and improve long-term oncologic outcomes. The patient’s serum β-hCG normalized after the first cycle of chemotherapy. Subsequently, she received 3 additional cycles of consolidation therapy.

Based on current evidence and insights from the present case, we propose the following management strategies for pulmonary ETT. First, in reproductive-aged women presenting with unexplained vaginal bleeding and mildly elevated β-hCG after exclusion of pregnancy, proactive chest imaging and timely surgical biopsy should be considered to establish a definitive diagnosis. Second, for isolated and completely resectable pulmonary lesions, fertility-sparing local excision is recommended, particularly in young patients. Third, the use of adjuvant chemotherapy should be individualized, with careful consideration of established high-risk pathological features balanced against potential treatment-related toxicities. Finally, the treatment of solid tumors is rapidly evolving. Novel systemic treatments, especially immunotherapy, have become transformative options for chemoresistant or high-risk GTN.^[21]^ In this context, managing pulmonary ETT is now closely aligned with precision medicine. Future studies should use multicenter data to develop and validate a strong risk-stratification algorithm which include reliable biomarkers and clinicopathological factors to better identify high-risk patients. Long-term follow-up is also needed to clarify the benefits and risks of adjuvant chemotherapy. Together, these efforts will create evidence-based protocols that optimize cancer outcomes while reducing overtreatment.

Current evidence regarding long-term prognosis and subsequent pregnancy outcomes in patients who achieve complete remission remains limited. The longest documented disease-free survival following pulmonary resection extends to 5 years without recurrence.^[16]^ To date, only 1 posttreatment pregnancy has been reported in a patient who conceived unexpectedly during estrogen replacement therapy for chemotherapy-induced ovarian failure. The patient delivered vaginally with postpartum normalization of serum β-hCG; however, long-term follow-up data remain unavailable.^[3]^ Given the potential risk of disease recurrence during or after pregnancy, and based on pregnancy outcome data from stage I uterine ETT patients treated with fertility-sparing approaches, some experts have proposed management strategies for pulmonary ETT patients wishing to preserve fertility. These include maintaining a complete remission interval of at least 3 years before attempting conception, with consideration of hysterectomy after pregnancy completion.^[15]^ It should be noted that these recommendations remain exploratory, as patients managed under this strategy have not received formal fertility counseling. Furthermore, both the optimal interval between treatment and conception and the necessity of prophylactic hysterectomy in patients with normalized β-hCG and no evidence of recurrence after pregnancy require further investigation.

Our patient achieved complete remission following initial treatment, with restoration of normal menstrual cycles and no evidence of disease recurrence throughout the 3-year follow-up period. The patient has expressed no current intention for pregnancy. However, should she become pregnant in the future, monitoring for disease recurrence would present significant challenges. Given that most patients with pulmonary ETT remain asymptomatic, and considering the limitations of CT/PET-CT imaging for surveillance of pulmonary lesions during pregnancy, there is a clear need to explore complementary diagnostic strategies for this special form of GTN. In this context, the integration of artificial intelligence (AI)-assisted methods in multimodal studies holds promise for improving diagnostic accuracy and enabling more precise risk stratification in such complex clinical settings.^[22,23]^ It is also important to note that the partial immunosuppression associated with pregnancy may potentially facilitate more rapid disease progression. Therefore, we emphasize the importance of comprehensive pre-pregnancy counseling to discuss these potential risks. We also advocate for intensified, multidisciplinary monitoring throughout any subsequent pregnancy to enable early detection of potential recurrence and to ensure appropriate management of both the pregnancy and any necessary treatment.

## 4. Conclusion

Collectively, available evidence suggests that fertility-preserving strategies may be a viable option for young patients with pulmonary ETT. Chemotherapy regimens should be individualized following a comprehensive assessment of risk factors. However, the rarity of the disease and the paucity of long-term survival data necessitate extended follow-up and the accumulation of more cases to validate these approaches. We strongly advocate for the implementation of structured follow-up protocols, which will be critical for refining future therapeutic strategies and guiding pregnancy management in this patient population.

## Acknowledgments

During the preparation of this work the authors used ChatGPT 4-o in order to improve language. After using this tool, the authors reviewed and edited the content as needed and take full responsibility for the content of publication.

## Author contributions

**Conceptualization:** Zhuo Yang, Qianqian Wang.

**Data curation:** Zhuo Yang, Shufang Chang.

**Formal analysis:** Zhuo Yang.

**Project administration:** Qianqian Wang.

**Resources:** Shufang Chang.

**Supervision:** Qianqian Wang.

**Writing – original draft:** Zhuo Yang.

**Writing – review & editing:** Zhuo Yang, Shufang Chang, Jiangchuan Sun, Qianqian Wang.

## Supplementary Material


