# Supplementary material for: The diagnostic and therapeutic challenges of pulmonary epithelioid trophoblastic tumor: A case report and literature review
Source: Medicine (Baltimore). 2026 Apr 10;105(15):e48248. doi: 10.1097/MD.0000000000048248 (PMC13592941; doi:10.1097/MD.0000000000048248)

Supplementary Figure. CT image of the patient's 3-year follow-up after lung lesion resection

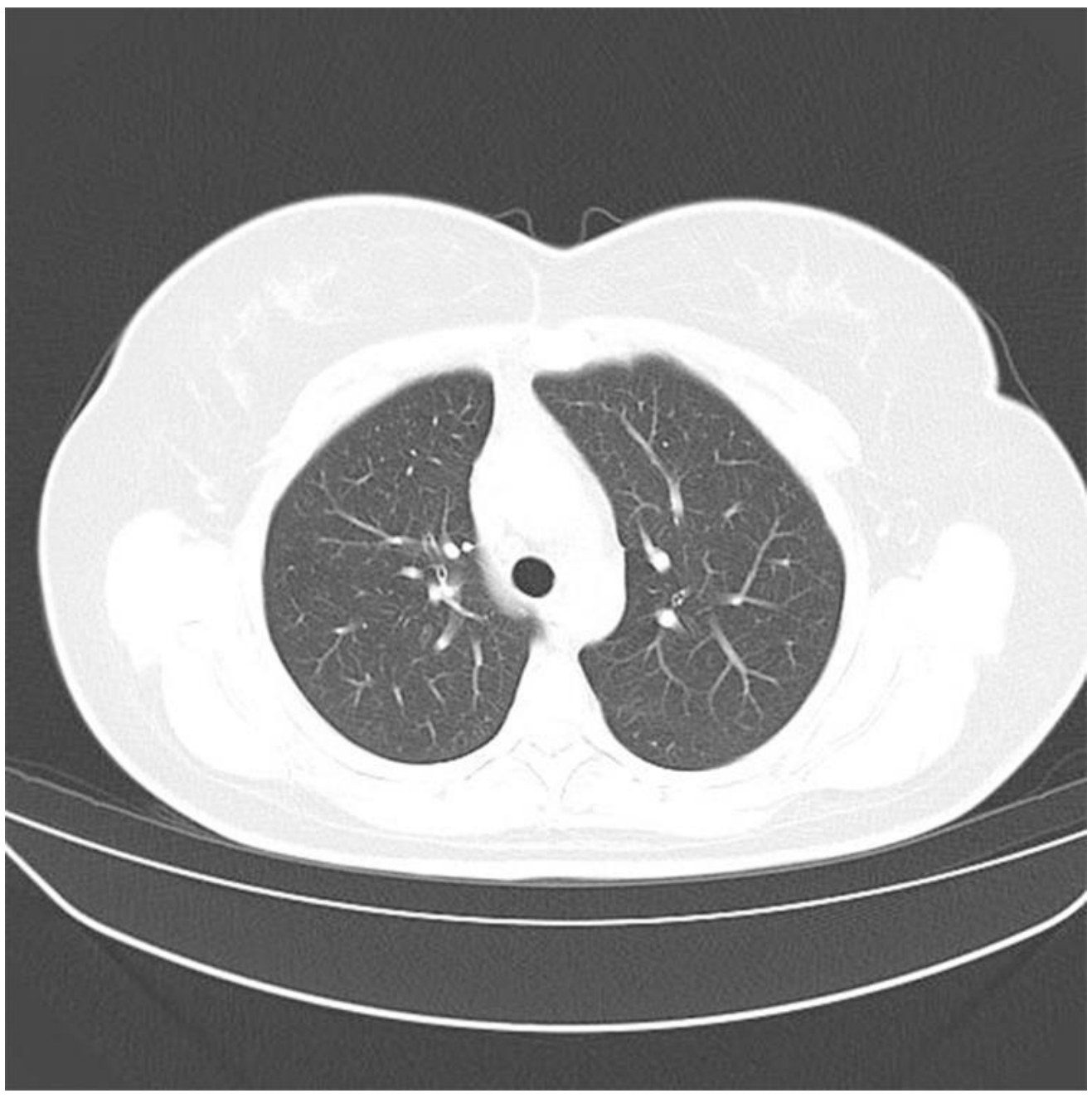

Supplement: Supplementary file 1 [file medi-105-e48248-s001.pdf]
